# Emerging T cell immunoregulatory mechanisms in multiple sclerosis and Alzheimer’s disease

**DOI:** 10.3389/fnagi.2024.1350240

**Published:** 2024-02-16

**Authors:** Daniel Hawiger

**Affiliations:** Department of Molecular Microbiology and Immunology, Saint Louis University School of Medicine, St. Louis, MO, United States

**Keywords:** multiple sclerosis, Alzheimer’s disease, tolerance, Tcells, dendritic cells

## Abstract

Multiple sclerosis (MS) and Alzheimer’s disease (AD) are neuroinflammatory and neurodegenerative diseases with considerable socioeconomic impacts but without definitive treatments. AD and MS have multifactorial pathogenesis resulting in complex cognitive and neurologic symptoms and growing evidence also indicates key functions of specific immune cells. Whereas relevant processes dependent on T cells have been elucidated in both AD and MS, mechanisms that can control such immune responses still remain elusive. Here, a brief overview of select recent findings clarifying immunomodulatory mechanisms specifically induced by tolerogenic dendritic cells to limit the activation and functions of neurodegenerative T cells is presented. These insights could become a foundation for new cutting-edge research as well as therapeutic strategies.

## The roles of T cells in multiple sclerosis and Alzheimer’s disease

Multiple sclerosis (MS) is a complex autoimmune disease characterized by inflammation of the central nervous system (CNS) following the attack of immune cells that destroy components of the myelin sheath surrounding the neuronal axons of the nerves, leading to demyelination and neurologic dysfunction. In most cases, MS is characterized by a relapsing–remitting disease course, although some patients with MS suffer from the primary-progressive form characterized by its steady progression. Many MS patients eventually develop a secondary-progressive MS characterized by a steady progression of symptoms (Dendrou et al., 2015; Baecher-Allan et al., 2018). Both genetic and environmental factors including past viral infections can contribute to the development of MS but the exact mechanisms resulting in MS pathogenesis in individual patients are unknown. Nevertheless, the processes directly leading to development of MS depend on an aberrant response of the immune system directed against specific antigens derived from components of the CNS (Hafler et al., 2007; Patsopoulos et al., 2011; Beecham et al., 2013; Lill et al., 2013). This autoimmune process can be initiated when specialized antigen presenting cells (APCs), including conventional dendritic cells (cDCs), activate autoreactive CD4^+^ T lymphocytes (cells). Such neural antigen-specific (encephalitogenic) CD4^+^ T cells can enter the CNS and be re-activated by the functions of microglia and possibly other of myeloid cells resulting in a recruitment of cytotoxic CD8^+^ T cells and establishing an inflammatory lesion (Kawakami et al., 2005; Raddassi et al., 2011; Jones and Hawiger, 2017; Absinta et al., 2021; Schnell et al., 2021; Lanz et al., 2022). Further, CD4^+^ T cells orchestrate functions of autoreactive B cells that, along with natural killer (NK) cells and macrophages, crucially contribute to MS immunopathology (Dendrou et al., 2015; Baecher-Allan et al., 2018).

The pathogenesis of another notorious neurologic disorder, Alzheimer’s disease (AD), remains elusive. AD is a devastating, progressive neurodegenerative disease affecting millions of people, including a large percentage of the population over 85 years old. Microscopic hallmarks of the disease are characterized by early deposition of amyloid-β (Aβ) plaques followed by formation of neurofibrillary tangles composed of hyperphosphorylated tau protein. Although deposition of Aβ is a necessary factor in AD pathogenesis, its accumulation appears as insufficient for neurodegeneration and cognitive decline. In contrast, pathological tau accumulation is closely linked with neurodegeneration and cognitive decline (DeMarshall et al., 2016; Chen et al., 2023). In contrast to MS and other autoimmune disease in which the key roles of T cells have been well established, T cells have been only recently implicated in pathogenesis of in AD. Previous results have suggested that T cells promote tissue pathology and cognitive defects in AD but these immune cells may also have beneficial functions such as clearing the amyloid plaques (Mietelska-Porowska and Wojda, 2017; Chen et al., 2023). The specific T cell antigens and the relevant functions of major T cell types including CD4^+^ T helper(Th)1, Th17, Th2, and CD8^+^ cytotoxic T cells, whose specific functions have been delineated in case of MS, remain less clear in AD. For example, Th1 cells were found to either exacerbate neuroinflammation or, conversely, had beneficial effects helping to clear amyloid plaques (Mietelska-Porowska and Wojda, 2017). Such contradictory results have been attributed to specific disease models used for research as well as various stages of a disease process at which corresponding roles of T cell were investigated. In contrast, most recent results obtained from a highly relevant AD tauopathy model have clearly implicated the role of pathogenic T cells and their specific effector cytokines in pthogensis of AD (Chen et al., 2023). These authors discovered relevant T cell adaptive immune responses, further supported by findings that T cells are present in the brain parenchyma from AD patients and that enrichment of T cells highly correlates with the severity of brain atrophy. Crucially, the inhibition of interferon-gamma, a major Th1 cytokine, significantly ameliorated brain atrophy. Further, a unique TCR clonal expansion of pathogenic T cells observed in this study is consistent with the underlying pathologies that may be shared with a similarly observed expansion of autoimmune T cells during MS (Cao et al., 2015; Hayashi et al., 2021; Chen et al., 2023). Further consistent with finding AD-associated neurodegenerative T cells in the CNS, such relevant T cells were also recently detected in peripheral blood from AD patients by the transcriptomic analysis that utilized a newly established Seqtometry platform based on direct profiling of gene expression (Kousnetsov et al., 2024). These results are also in agreement with the known paths of T cell circulation between CNS and the peripheral immune system (Kawakami et al., 2005; Raddassi et al., 2011; Jones and Hawiger, 2017; Absinta et al., 2021; Schnell et al., 2021; Lanz et al., 2022). Overall, the regulation of T cell functions appears as a critical objective for achieving successful immunotherapies in case of both AD and MS.

## The indispensable roles of pT_REGS_ and conventional dendritic cells in immunoregulation

The T cell-driven neuroinflammation is susceptible to a control by Foxp3^+^ regulatory CD4^+^ T cells (T_REGS_) (Jones and Hawiger, 2017). Accordingly, T_REGS_ have been established to play a pivotal role in protection and recovery from MS and its animal models by suppressing relevant autoreactive T cells (O'Connor and Anderton, 2008; Lowther and Hafler, 2012; Kleinewietfeld and Hafler, 2014). The functions of T_REGS_ in AD are still being elucidated and a depletion of T_REGS_ resulted in either blocking or promoting the AD disease process (Mietelska-Porowska and Wojda, 2017). A key recent study uncovered that increased numbers of T_REGS_ indeed correlated with the decreased disease severity, further establishing the beneficial roles of T_REGS_ in the AD process (Chen et al., 2023). In MS models, early pioneering studies showed that T cell receptor (TCR)-transgenic mice specific for myelin basic protein (MBP), a major neural antigen, succumbed to experimental autoimmune encephalitis (EAE), a mouse model of MS, when such animals were crossed onto a genetic background that precluded a development of T_REGS_ while allowing a development of autoreactive T cells (Lafaille et al., 1994). Consistently, a depletion of T_REGS_ *in vivo* by anti-CD25 antibody also exacerbated EAE (Kohm et al., 2006). Based on these early insights, therapies focused on functions of T_REGS_ have been proposed as an approach to block neuroinflammation (O'Connor and Anderton, 2008; Spence et al., 2015; Cheng et al., 2017).

Foxp3-expressing T_REGS_ are a heterogenous group of T cells that includes thymically derived (t)T_REGS_ developing in the thymus. Whereas functions of such tT_REGS_ are indispensable for the maintenance of immune homeostasis, they are not sufficient to prevent an initiation of specific autoimmune process such as in EAE. Instead, mechanisms blocking antigen-specific autoimmunity rely on other Foxp3-expressing T_REGS_ that are induced *de novo* outside the thymus from T cells in response to corresponding self-antigens (Iberg et al., 2017; Jones and Hawiger, 2017). By focusing on relevant antigens, such peripherally induced (p)T_REGS_ can efficiently limit harmful effector autoimmune responses (Kretschmer et al., 2005; Iberg et al., 2017). Therefore, a *de novo* induction of antigen-specific peripheral pT_REGS_ critically complements the functions of tT_REGS_, and has a major role in shaping the immune homeostasis. Experimental results in animal disease models confirmed that induction and functions of pT_REGS_ were required for blocking neurologic symptoms in EAE despite the normal presence of thymically-derived tT_REGS_ (Jones et al., 2015, 2016; Jones and Hawiger, 2017). Similar findings were also extended to some other autoimmune disease models (Iberg and Hawiger, 2020a; Bourque and Hawiger, 2022a).

Given their importance, the induction of pT_REGS_ from naive T cells must be carefully regulated. A key role in this processes is performed by specialized cDCs that can present antigens in the tolerogenic context that depends on a specific molecular crosstalk between cDCs and T cells to skew the actively induced T cell differentiation toward pT_REGS_ instead of priming effector responses (Iberg and Hawiger, 2020a). Overall, the versatility of T cell differentiation depends on comprehensive characteristics of cDCs which represent a heterogenous group of cells including two major types 1 and 2 (cDC1 and cDC2) that differ in their specific development, phenotypes, and immune functions (Durai and Murphy, 2016; Murphy et al., 2016). Whereas all cDCs excel at presentation of antigens to T cells, the CCR7^+^ migratory cDCs can deliver various peripherally-acquired antigens to lymph nodes (LNs) (Bourque and Hawiger, 2022a). Such cDCs also express immunoregulatory molecules including PD-L1, PD-L2, and CD200, and can promote relevant T cell tolerance in the case of multiple antigens (Leventhal et al., 2016; Maier et al., 2020). However, migratory cDCs also have key roles in the initiation of effector responses (Scheinecker et al., 2002; GeurtsvanKessel et al., 2008; Kim et al., 2010; Bajana et al., 2012; Krishnaswamy et al., 2018; Jenkins et al., 2021). In contrast, other types of cDCs present in lymphoid organs such as LNs and spleen, specialize in promoting induction of pT_REGS_ with anti-autoimmune roles (Iberg and Hawiger, 2020a; Bourque and Hawiger, 2022a). These pT_REGS_-inducing cDC1s are characterized by high expression of immunomodulatory molecules B and T lymphocyte associated/attenuator (BTLA) and T-cell immunoglobulin mucin-3 (TIM-3) (Kim et al., 2020; Bourque and Hawiger, 2022a; Tang et al., 2022). The constitutive expression of these pro-tolerogenic immunomodulators, as part of the pre-determined developmental program, provides BTLA^hi^ cDC1s with inherent capabilities to facilitate induction of pT_REGS_ from T cells activated in response to various antigens presented by these cDC1s (Bourque and Hawiger, 2019, 2022a; Iberg and Hawiger, 2020a). Specifically, through interactions with herpesvirus entry mediator (HVEM) expressed in naive T cells, BTLA increase expression of CD5 in T cells (Jones et al., 2016; Bourque and Hawiger, 2019). This BTLA-HVEM-CD5 immunomodulatory axis facilitates the pT_REGS_ conversion from naive T cells by decreasing the sensitivity of T cells to various pro-inflammatory cytokines through limiting functions of mammalian target of rapamycin (mTOR) (Chen et al., 2003; Henderson et al., 2015; Jones et al., 2016; Bourque and Hawiger, 2019). Therefore, tolerogenic functions of BTLA^hi^ cDC1s also crucially rely on other mechanisms notably including the production and activation of transforming growth factor beta (TGF-β), well established as critically required for the *de novo* expression of *Foxp3* in developing pT_REGS_ (Chen et al., 2003; Bourque and Hawiger, 2018). Additional mechanisms such as those dependent on indoleamine 2,3-dioxygenase (IDO) and aryl hydrocarbon receptor (AHR) also can play important roles in these tolerogenic processes (Bourque and Hawiger, 2018). In the absence of specific tolerogenic functions of BTLA^hi^ cDC1s, the *de novo* induction of pT_REGS_ controlling specific autoimmune responses is compromised, as observed using either *Btla* genetic deletion or a treatment with anti-BTLA blocking antibodies in mouse models *in vivo* (Jones et al., 2016; Iberg et al., 2017, 2022). Nevertheless, while IDO may complement tolerogenic functions of BTLA^hi^ cDC1s, recent results have also shown IDO activation in some mature cDC1s with high CCR7 expression, and not corresponding to BTLA^hi^ cDC1s, that may promote the tolerogenic functionality in autoimmune models including non-obese diabetes (NOD) and EAE (Price et al., 2015; Tabansky et al., 2018; Gargaro et al., 2022).

The understanding of the tolerogenic roles of cDCs comes mostly from studies that focused on these functions among the cDCs present in the peripheral immune system, outside the CNS. However, because T cells trafficking to the CNS also recirculate to the peripheral immune system where they can be maintained and activated (Kawakami et al., 2005; Raddassi et al., 2011; Jones and Hawiger, 2017; Absinta et al., 2021; Schnell et al., 2021; Lanz et al., 2022), the tolerogenic mechanisms initiated in the periphery extend to the control of the pathogenic immune processes in the CNS (Jones et al., 2015; Jones and Hawiger, 2017). Therefore, not only are the molecular mechanisms underlying the tolerogenic roles of cDCs of acute research interest, but they could also become potential therapeutic targets.

## Tolerogenic mechanisms under pro-inflammatory conditions

The *in vivo* targeted delivery of self-antigens to BTLA^hi^ cDC1 utilizing recombinant antibodies and other reagents binding to specific surface receptors on these cells represents a key pro-tolerogenic strategy (Iberg and Hawiger, 2019, 2020b; Bourque and Hawiger, 2021, 2022b). However, the full realization of such therapeutic potentials, especially for translationally relevant settings, still remains limited by the complexities of molecular mechanisms within the cDCs. Recent results have revealed that even under steady conditions (in the absence of specific, disease-associated pro-inflammatory signals), some cDCs induce CD4^+^ T cells with an increased potential for autoimmune differentiation (Opejin et al., 2020; Bourque and Hawiger, 2022a). In EAE models such “pre-effectors” can readily convert into encephalitogenic effector T cells resulting in a development of autoimmune disease. Therefore, a careful consideration is required when selecting specific targets and routes for antigen delivery to cDCs with intended pro-tolerogenic effects (Bourque and Hawiger, 2022a,b).

Recent findings also uncovered that tolerogenic BTLA^hi^ cDC1s are highly susceptible to pro-inflammatory conditions that result in ablation of these cells through an acute cell death mediated by TNF-α (Iberg et al., 2022; Bourque and Hawiger, 2023a). TNF-α is released by cDCs, T cells, and other types of immune cells upon their initial immune activation (Bourque and Hawiger, 2023b). TNF-α has been shown in numerous mouse models to be crucially involved in a propagation of the maturation (activation) of cDCs (Bardou et al., 2021; Cabeza-Cabrerizo et al., 2021). Consistently, TNF-α is involved in the pathogenesis of several autoimmune and pro-inflammatory disease in humans including AD and MS and also others such as Crohn’s disease and arthritis (Chang et al., 2017; Jang et al., 2021). Significantly, the levels of TNF-α are increased in blood of patients with AD, and clinical and animal studies have demonstrated a link between excess TNF-α levels in the brain and AD development (Chang et al., 2017). At the same time, anti-TNF-α treatments have been only partially successful, and their lack of effectiveness, particularly in case of MS, can likely be attributed to complex signaling pathways that are initiated by TNF-α sensed by two different receptors, tumor necrosis factor receptor 1 (TNFR1) and TNFR2 (Bourque and Hawiger, 2023b). The expression patterns and functions of these receptors differ depending on the cell type. Both receptors can respond to TNF-α bound to a membrane but only TNFR1 can proficiently respond to TNF-α in its soluble form (Wajant and Siegmund, 2019). TNFR1 is expressed by nearly all cell types, whereas TNFR2 expression is restricted to regulatory T cells, myeloid cells, glial cells, certain endothelial cells, and is also induced by certain T and B cell subsets, epithelial cells, and fibroblasts (Wajant and Siegmund, 2019). Both receptors activate proinflammatory pathways such as the NF-κB (Wajant and Siegmund, 2019). TNFR1 induces necroptotic and apoptotic cell death through complex processes dependent on receptor-interacting serine/threonine-protein kinase 3 (RIPK3) and caspase 8, respectively (Wajant and Siegmund, 2019). These diverse mechanisms mediated by TNF-α underscore the pleiotropic outcomes of the sensing of this cytokine by cDCs that interact with different types of effector and regulatory T cells. Recent results have elucidated that, among all cDCs, the highest expression of TNFR1 characterizes tolerogenic BTLA^hi^ cDC1s (Iberg et al., 2022). Therefore, under pro-inflammatory conditions the BTLA^hi^ cDC1s die rapidly in response to the released TNF-α whose copious amounts are also produced in the autocrine/paracrine fashion by these cells (Iberg et al., 2022). Although the ablation of BTLA^hi^ cDC1s is only transient, with the populations of cDCs returning to their homeostatic numbers within a week, the acute absence of these tolerogenic cDCs shifts a balance away from the induction of pT_REGS_ (Iberg et al., 2022; Bourque and Hawiger, 2023b). Such conditions blocking pro-tolerogenic mechanisms can be instead more conducive to differentiation and function of potentially pathogenic effector T cells (Bourque and Hawiger, 2023b). Therefore, designs of new therapies focused on either induction or restoration/enhancement of tolerogenic mechanisms must also account for a loss of cDCs with tolerogenic functions occurring under conditions that underly various pro-inflammatory diseases.

## Conclusions and future directions

It is becoming increasingly clear that specific immunoregulatory mechanisms can limit the disease process in MS, and possibly also in AD. Whereas it is still not possible to fully utilize such processes for translationally relevant clinical applications, recent insights into the tolerogenic mechanisms of dendritic cells as well as the impact of key pro-inflammatory mediators on these functions, could pave the way for designing effective strategies that could better counter pathologies underlying MS and AD. Further, new tools may facilitate a detection of neurodegenerative T cells in AD and MS patients for early diagnostic as well as monitoring therapeutic outcomes. Overall, the future advances will lead to designing targeted and antigen-specific immunotherapies to fully utilize the potential of regulatory T cell mechanisms.

## Author contributions

DH: Conceptualization, Writing – original draft, Writing – review & editing.

## References

[ref1] Absinta M. Maric D. Gharagozloo M. Garton T. Smith M. D. Jin J.. (2021). A lymphocyte-microglia-astrocyte axis in chronic active multiple sclerosis. Nature 597, 709–714. doi: 10.1038/s41586-021-03892-7, PMID: 34497421 PMC8719282

[ref2] Baecher-Allan C. Kaskow B. J. Weiner H. L. (2018). Multiple sclerosis: mechanisms and immunotherapy. Neuron 97, 742–768. doi: 10.1016/j.neuron.2018.01.021 29470968

[ref3] Bajana S. Roach K. Turner S. Paul J. Kovats S. (2012). IRF4 promotes cutaneous dendritic cell migration to lymph nodes during homeostasis and inflammation. J. Immunol. 189, 3368–3377. doi: 10.4049/jimmunol.1102613, PMID: 22933627 PMC3448873

[ref4] Bardou M. Postat J. Loaec C. Lemaitre F. Ronteix G. Garcia Z.. (2021). Quorum sensing governs collective dendritic cell activation in vivo. EMBO J. 40:e107176. doi: 10.15252/embj.2020107176, PMID: 34124789 PMC8327941

[ref5] Beecham A. H. Patsopoulos N. A. Xifara D. K. Davis M. F. Kemppinen A. Cotsapas C.. (2013). Analysis of immune-related loci identifies 48 new susceptibility variants for multiple sclerosis. Nat. Genet. 45, 1353–1360. doi: 10.1038/ng.2770, PMID: 24076602 PMC3832895

[ref6] Bourque J. Hawiger D. (2018). Immunomodulatory bonds of the partnership between dendritic cells and T cells. Crit. Rev. Immunol. 38, 379–401. doi: 10.1615/CritRevImmunol.2018026790, PMID: 30792568 PMC6380512

[ref7] Bourque J. Hawiger D. (2019). The BTLA–HVEM–CD5 Immunoregulatory Axis–an instructive mechanism governing pTreg cell differentiation. Front. Immunol. 10:1163. doi: 10.3389/fimmu.2019.01163, PMID: 31191536 PMC6541033

[ref8] Bourque J. Hawiger D. (2021). Current and future immunotherapies for multiple sclerosis. Mo. Med. 118, 334–339. PMID: 34373668 PMC8343631

[ref9] Bourque J. Hawiger D. (2022a). Variegated outcomes of T cell activation by dendritic cells in the steady state. J. Immunol. 208, 539–547. doi: 10.4049/jimmunol.2100932, PMID: 35042789 PMC8852351

[ref10] Bourque J. Hawiger D. (2022b). Applications of antibody-based antigen delivery targeted to dendritic cells in vivo. Antibodies (Basel) 11:10008. doi: 10.3390/antib11010008 PMC8884005 35225867

[ref11] Bourque J. Hawiger D. (2023a). Life and death of tolerogenic dendritic cells. Trends Immunol. 44, 110–118. doi: 10.1016/j.it.2022.12.006, PMID: 36599743 PMC9892261

[ref12] Bourque J. Hawiger D. (2023b). Activation, amplification, and ablation as dynamic mechanisms of dendritic cell maturation. Biology (Basel) 12:716. doi: 10.3390/biology12050716 37237529 PMC10215461

[ref13] Cabeza-Cabrerizo M. Cardoso A. Minutti C. M. Pereira da Costa M. Reis E. S. C. (2021). Dendritic cells revisited. Annu. Rev. Immunol. 39, 131–166. doi: 10.1146/annurev-immunol-061020-053707 33481643

[ref14] Cao Y. Goods B. A. Raddassi K. Nepom G. T. Kwok W. W. Love J. C.. (2015). Functional inflammatory profiles distinguish myelin-reactive T cells from patients with multiple sclerosis. Sci. Transl. Med. 7:287ra74. doi: 10.1126/scitranslmed.aaa8038 PMC4497538 25972006

[ref15] Chang R. Yee K. L. Sumbria R. K. (2017). Tumor necrosis factor alpha inhibition for Alzheimer's disease. J. Cent. Nerv. Syst. Dis. 9:1179573517709278. doi: 10.1177/1179573517709278 28579870 PMC5436834

[ref16] Chen X. Firulyova M. Manis M. Herz J. Smirnov I. Aladyeva E.. (2023). Microglia-mediated T cell infiltration drives neurodegeneration in tauopathy. Nature 615, 668–677. doi: 10.1038/s41586-023-05788-0, PMID: 36890231 PMC10258627

[ref17] Chen W. Jin W. Hardegen N. Lei K. J. Li L. Marinos N.. (2003). Conversion of peripheral CD4+CD25- naive T cells to CD4+CD25+ regulatory T cells by TGF-beta induction of transcription factor Foxp3. J. Exp. Med. 198, 1875–1886. doi: 10.1084/jem.20030152, PMID: 14676299 PMC2194145

[ref18] Cheng Y. Sun L. Xie Z. Fan X. Cao Q. Han J.. (2017). Diversity of immune cell types in multiple sclerosis and its animal model: pathological and therapeutic implications. J. Neurosci. Res. 95, 1973–1983. doi: 10.1002/jnr.24023, PMID: 28084640 PMC5573979

[ref19] DeMarshall C. A. Nagele E. P. Sarkar A. Acharya N. K. Godsey G. Goldwaser E. L.. (2016). Detection of Alzheimer's disease at mild cognitive impairment and disease progression using autoantibodies as blood-based biomarkers. Alzheimer’s Dement. 3, 51–62. doi: 10.1016/j.dadm.2016.03.002, PMID: 27239548 PMC4879649

[ref20] Dendrou C. A. Fugger L. Friese M. A. (2015). Immunopathology of multiple sclerosis. Nat. Rev. Immunol. 15, 545–558. doi: 10.1038/nri3871 26250739

[ref21] Durai V. Murphy K. M. (2016). Functions of murine dendritic cells. Immunity 45, 719–736. doi: 10.1016/j.immuni.2016.10.010, PMID: 27760337 PMC5145312

[ref22] Gargaro M. Scalisi G. Manni G. Briseño C. G. Bagadia P. Durai V.. (2022). Indoleamine 2,3-dioxygenase 1 activation in mature cDC1 promotes tolerogenic education of inflammatory cDC2 via metabolic communication. Immunity 55, 1032–1050.e14. doi: 10.1016/j.immuni.2022.05.013, PMID: 35704993 PMC9220322

[ref23] GeurtsvanKessel C. H. Willart M. A. M. van Rijt L. S. Muskens F. Kool M. Baas C.. (2008). Clearance of influenza virus from the lung depends on migratory langerin+CD11b− but not plasmacytoid dendritic cells. J. Exp. Med. 205, 1621–1634. doi: 10.1084/jem.20071365, PMID: 18591406 PMC2442640

[ref24] Hafler D. A. Compston A. Sawcer S. Lander E. S. Daly M. J. De Jager P. L.. (2007). Risk alleles for multiple sclerosis identified by a genomewide study. N. Engl. J. Med. 357, 851–862. doi: 10.1056/NEJMoa073493 17660530

[ref25] Hayashi F. Isobe N. Glanville J. Matsushita T. Maimaitijiang G. Fukumoto S.. (2021). A new clustering method identifies multiple sclerosis-specific T-cell receptors. Ann. Clin. Transl. Neurol. 8, 163–176. doi: 10.1002/acn3.51264, PMID: 33400858 PMC7818280

[ref26] Henderson J. G. Opejin A. Jones A. Gross C. Hawiger D. (2015). CD5 instructs Extrathymic regulatory T cell development in response to self and Tolerizing antigens. Immunity 42, 471–483. doi: 10.1016/j.immuni.2015.02.010 25786177

[ref27] Iberg C. A. Bourque J. Fallahee I. Son S. Hawiger D. (2022). TNF-α sculpts a maturation process in vivo by pruning tolerogenic dendritic cells. Cell Rep. 39:110657. doi: 10.1016/j.celrep.2022.110657, PMID: 35417681 PMC9113652

[ref28] Iberg C. A. Hawiger D. (2019). Advancing immunomodulation by in vivo antigen delivery to DEC-205 and other cell surface molecules using recombinant chimeric antibodies. Int. Immunopharmacol. 73, 575–580. doi: 10.1016/j.intimp.2019.05.037, PMID: 31228685 PMC6707533

[ref29] Iberg C. A. Hawiger D. (2020a). Natural and induced Tolerogenic dendritic cells. J. Immunol. 204, 733–744. doi: 10.4049/jimmunol.1901121, PMID: 32015076 PMC7006631

[ref30] Iberg C. A. Hawiger D. (2020b). Targeting dendritic cells with antigen-delivering antibodies for amelioration of autoimmunity in animal models of multiple sclerosis and other autoimmune diseases. Antibodies (Basel) 9:23. doi: 10.3390/antib9020023, PMID: 32549343 PMC7345927

[ref31] Iberg C. A. Jones A. Hawiger D. (2017). Dendritic cells as inducers of peripheral tolerance. Trends Immunol. 38, 793–804. doi: 10.1016/j.it.2017.07.007, PMID: 28826942 PMC5669994

[ref32] Jang D. I. Lee A. H. Shin H. Y. Song H. R. Park J. H. Kang T. B.. (2021). The role of tumor necrosis factor alpha (TNF-alpha) in autoimmune disease and current TNF-alpha inhibitors in therapeutics. Int. J. Mol. Sci. 22:2719. doi: 10.3390/ijms22052719, PMID: 33800290 PMC7962638

[ref33] Jenkins M. M. Bachus H. Botta D. Schultz M. D. Rosenberg A. F. Leon B.. (2021). Lung dendritic cells migrate to the spleen to prime long-lived TCF1(hi) memory CD8(+) T cell precursors after influenza infection. Sci. Immunol. 6:eabg6895. doi: 10.1126/sciimmunol.abg6895, PMID: 34516781 PMC8559272

[ref34] Jones A. Bourque J. Kuehm L. Opejin A. Teague R. M. Gross C.. (2016). Immunomodulatory functions of BTLA and HVEM govern induction of Extrathymic regulatory T cells and tolerance by dendritic cells. Immunity 45, 1066–1077. doi: 10.1016/j.immuni.2016.10.008, PMID: 27793593 PMC5112132

[ref35] Jones A. Hawiger D. (2017). Peripherally induced regulatory T cells: recruited protectors of the central nervous system against autoimmune Neuroinflammation. Front. Immunol. 8:532. doi: 10.3389/fimmu.2017.00532, PMID: 28536579 PMC5422564

[ref36] Jones A. Opejin A. Henderson J. G. Gross C. Jain R. Epstein J. A.. (2015). Peripherally induced tolerance depends on peripheral regulatory T cells that require Hopx to inhibit intrinsic IL-2 expression. J. Immunol. 195, 1489–1497. doi: 10.4049/jimmunol.1500174, PMID: 26170384 PMC4530038

[ref37] Kawakami N. Odoardi F. Ziemssen T. Bradl M. Ritter T. Neuhaus O.. (2005). Autoimmune CD4+ T cell memory: lifelong persistence of encephalitogenic T cell clones in healthy immune repertoires. J. Immunol. 175, 69–81. doi: 10.4049/jimmunol.175.1.69, PMID: 15972633

[ref38] Kim S. Bagadia P. Anderson D. A. Liu T.-T. Huang X. Theisen D. J.. (2020). High amount of transcription factor IRF8 engages AP1-IRF composite elements in enhancers to direct type 1 conventional dendritic cell identity. Immunity 53, 759–774.e9. doi: 10.1016/j.immuni.2020.07.018, PMID: 32795402 PMC8193644

[ref39] Kim T. S. Hufford M. M. Sun J. Fu Y.-X. Braciale T. J. (2010). Antigen persistence and the control of local T cell memory by migrant respiratory dendritic cells after acute virus infection. J. Exp. Med. 207, 1161–1172. doi: 10.1084/jem.20092017, PMID: 20513748 PMC2882836

[ref40] Kleinewietfeld M. Hafler D. A. (2014). Regulatory T cells in autoimmune neuroinflammation. Immunol. Rev. 259, 231–244. doi: 10.1111/imr.12169, PMID: 24712469 PMC3990868

[ref41] Kohm A. P. McMahon J. S. Podojil J. R. Begolka W. S. DeGutes M. Kasprowicz D. J.. (2006). Cutting edge: anti-CD25 monoclonal antibody injection results in the functional inactivation, not depletion, of CD4+CD25+ T regulatory cells. J. Immunol. 176, 3301–3305. doi: 10.4049/jimmunol.176.6.3301, PMID: 16517695

[ref42] Kousnetsov R. Bourque J. Surnov A. Fallahee I. Hawiger D. (2024). Single-cell sequencing analysis within biologically relevant dimensions. Cell Syst. 15, 83–103.e11. doi: 10.1016/j.cels.2023.12.005, PMID: 38198894

[ref43] Kretschmer K. Apostolou I. Hawiger D. Khazaie K. Nussenzweig M. C. von Boehmer H. (2005). Inducing and expanding regulatory T cell populations by foreign antigen. Nat. Immunol. 6, 1219–1227. doi: 10.1038/ni1265, PMID: 16244650

[ref44] Krishnaswamy J. K. Alsen S. Yrlid U. Eisenbarth S. C. Williams A. (2018). Determination of T follicular helper cell fate by dendritic cells. Front. Immunol. 9:2169. doi: 10.3389/fimmu.2018.02169, PMID: 30319629 PMC6170619

[ref45] Lafaille J. J. Nagashima K. Katsuki M. Tonegawa S. (1994). High incidence of spontaneous autoimmune encephalomyelitis in immunodeficient anti-myelin basic protein T cell receptor transgenic mice. Cell 78, 399–408. doi: 10.1016/0092-8674(94)90419-7, PMID: 7520367

[ref46] Lanz T. V. Brewer R. C. Ho P. P. Moon J. S. Jude K. M. Fernandez D.. (2022). Clonally expanded B cells in multiple sclerosis bind EBV EBNA1 and GlialCAM. Nature 603, 321–327. doi: 10.1038/s41586-022-04432-7, PMID: 35073561 PMC9382663

[ref47] Leventhal D. S. Gilmore D. C. Berger J. M. Nishi S. Lee V. Malchow S.. (2016). Dendritic cells coordinate the development and homeostasis of organ-specific regulatory T cells. Immunity 44, 847–859. doi: 10.1016/j.immuni.2016.01.025, PMID: 27037189 PMC4842258

[ref48] Lill C. M. Schjeide B. M. Graetz C. Ban M. Alcina A. Ortiz M. A.. (2013). MANBA, CXCR5, SOX8, RPS6KB1 and ZBTB46 are genetic risk loci for multiple sclerosis. Brain 136, 1778–1782. doi: 10.1093/brain/awt101, PMID: 23739915 PMC3673463

[ref49] Lowther D. E. Hafler D. A. (2012). Regulatory T cells in the central nervous system. Immunol. Rev. 248, 156–169. doi: 10.1111/j.1600-065X.2012.01130.x 22725960

[ref50] Maier B. Leader A. M. Chen S. T. Tung N. Chang C. LeBerichel J.. (2020). A conserved dendritic-cell regulatory program limits antitumour immunity. Nature 580, 257–262. doi: 10.1038/s41586-020-2134-y, PMID: 32269339 PMC7787191

[ref51] Mietelska-Porowska A. Wojda U. (2017). T lymphocytes and inflammatory mediators in the interplay between brain and blood in Alzheimer's disease: potential pools of new biomarkers. J. Immunol. Res. 2017:4626540. doi: 10.1155/2017/4626540 28293644 PMC5331319

[ref52] Murphy T. L. Grajales-Reyes G. E. Wu X. Tussiwand R. Briseno C. G. Iwata A.. (2016). Transcriptional control of dendritic cell development. Annu. Rev. Immunol. 34, 93–119. doi: 10.1146/annurev-immunol-032713-120204, PMID: 26735697 PMC5135011

[ref53] O'Connor R. A. Anderton S. M. (2008). Foxp3+ regulatory T cells in the control of experimental CNS autoimmune disease. J. Neuroimmunol. 193, 1–11. doi: 10.1016/j.jneuroim.2007.11.016 18077005

[ref54] Opejin A. Surnov A. Misulovin Z. Pherson M. Gross C. Iberg C. A.. (2020). A two-step process of effector programming governs CD4(+) T cell fate determination induced by antigenic activation in the steady state. Cell Rep. 33:108424. doi: 10.1016/j.celrep.2020.108424, PMID: 33238127 PMC7714042

[ref55] Patsopoulos N. A. Bayer Pharma MS Genetics Working Group, Steering Committees of Studies Evaluating IFNβ-1b and a CCR1-Antagonist; ANZgene Consortium; GeneMSA; International Multiple Sclerosis Genetics Consortium Esposito F. Reischl J. Esposito F. Reischl J.. (2011). Genome-wide meta-analysis identifies novel multiple sclerosis susceptibility loci. Ann. Neurol. 70, 897–912. doi: 10.1002/ana.22609 22190364 PMC3247076

[ref56] Price J. D. Hotta-Iwamura C. Zhao Y. Beauchamp N. M. Tarbell K. V. (2015). DCIR2+ cDC2 DCs and Zbtb32 restore CD4+ T-cell tolerance and inhibit diabetes. Diabetes 64, 3521–3531. doi: 10.2337/db14-1880, PMID: 26070317 PMC4587633

[ref57] Raddassi K. Kent S. C. Yang J. Bourcier K. Bradshaw E. M. Seyfert-Margolis V.. (2011). Increased frequencies of myelin oligodendrocyte glycoprotein/MHC class II-binding CD4 cells in patients with multiple sclerosis. J. Immunol. 187, 1039–1046. doi: 10.4049/jimmunol.1001543, PMID: 21653833 PMC3131477

[ref58] Scheinecker C. McHugh R. Shevach E. M. Germain R. N. (2002). Constitutive presentation of a natural tissue autoantigen exclusively by dendritic cells in the draining lymph node. J. Exp. Med. 196, 1079–1090. doi: 10.1084/jem.20020991, PMID: 12391019 PMC2194046

[ref59] Schnell A. Huang L. Singer M. Singaraju A. Barilla R. M. Regan B. M. L.. (2021). Stem-like intestinal Th17 cells give rise to pathogenic effector T cells during autoimmunity. Cell 184, 6281–6298.e23. doi: 10.1016/j.cell.2021.11.018, PMID: 34875227 PMC8900676

[ref60] Spence A. Klementowicz J. E. Bluestone J. A. Tang Q. (2015). Targeting Treg signaling for the treatment of autoimmune diseases. Curr. Opin. Immunol. 37, 11–20. doi: 10.1016/j.coi.2015.09.002, PMID: 26432763 PMC4679451

[ref61] Tabansky I. Keskin D. B. Watts D. Petzold C. Funaro M. Sands W.. (2018). Targeting DEC-205(−)DCIR2(+) dendritic cells promotes immunological tolerance in proteolipid protein-induced experimental autoimmune encephalomyelitis. Mol. Med. 24:17. doi: 10.1186/s10020-018-0017-6, PMID: 30134798 PMC6016871

[ref62] Tang R. Acharya N. Subramanian A. Purohit V. Tabaka M. Hou Y.. (2022). Tim-3 adapter protein Bat3 acts as an endogenous regulator of tolerogenic dendritic cell function. Sci. Immunol. 7:eabm0631. doi: 10.1126/sciimmunol.abm0631, PMID: 35275752 PMC9273260

[ref63] Wajant H. Siegmund D. (2019). TNFR1 and TNFR2 in the control of the life and death balance of macrophages. Front. Cell Dev. Biol. 7:91. doi: 10.3389/fcell.2019.00091, PMID: 31192209 PMC6548990

